# Immune checkpoint inhibitor induced neurocognitive deficits in patients

**DOI:** 10.1093/braincomms/fcad186

**Published:** 2023-06-20

**Authors:** Robert Zeiser, Marco Prinz

**Affiliations:** Department of Internal Medicine I, Medical Center—University of Freiburg, Faculty of Medicine, University of Freiburg, Freiburg, Germany; German Cancer Consortium (DKTK) Partner Site Freiburg, German Cancer Research Center (DKFZ), Heidelberg, Germany; Signalling Research Centres BIOSS and CIBSS—Centre for Integrative Biological Signalling Studies, University of Freiburg, Germany; Signalling Research Centres BIOSS and CIBSS—Centre for Integrative Biological Signalling Studies, University of Freiburg, Germany; Institute for Neuropathology, Medical Faculty, University of Freiburg, Germany; Center for Neuro Modulation, Faculty of Medicine, University of Freiburg, FreiburgGermany

## Abstract

This scientific commentary refers to ‘Neurological outcomes in immune checkpoint inhibitor-related neurotoxicity’, by Farina *et al*. (https://doi.org/10.1093/braincomms/fcad169)

**This scientific commentary refers to ‘Neurological outcomes in immune checkpoint inhibitor-related neurotoxicity’, by Farina *et al*. (https://doi.org/10.1093/braincomms/fcad169)**

Immune-related adverse events (irAE) that occur after immune checkpoint inhibitor therapy (ICI) can affect various organs including the peripheral (PNS) and central nervous systems (CNS). Treatment of irAEs includes interruption of ICI-therapy and administration of glucocorticosteroids as well as multiple experimental approaches.^1-3^ The frequency of neurological symptoms in patients receiving ICI ranges between 1% and 12%^4-6^, and the clinical picture includes meningitis, Guillain–Barré syndrome, hypophysitis, encephalitis, cranial and peripheral neuropathies and myasthenia gravis.^6,7^ In their article in *Brain Communications*, Farina *et al*.^8^ report on the outcomes of neurological irAEs in 147 patients with the intention to detect prognostic factors. The study included patients suffering from neurological irAEs at least grade ≥2 derived from two clinical networks (French Reference Center for Paraneoplastic Neurological Syndromes, Lyon, and OncoNeuroTox, Paris) during a period of 5 years,^8^ Neurological irAEs affected the PNS in 59.2% and the CNS in 34.7%, respectively.^8^ The authors found that the rate of changeover from severe to minor disability was higher in patients suffering from melanoma compared with lung cancer.^8^ Similarly, the disease manifestations myositis and neuromuscular junction disorders were connected to a higher rate of changeover from severe to minor disability.^8^ Conversely, neurological improvement was lower when patients were older or had paraneoplastic-like syndromes. The findings are clinically important as they indicate that neurological recovery from ICI-induced PNS, and CNS damage is connected to baseline characteristics of the patients’ malignancy and age, which may help to guide treatment decision in future clinical studies, e.g. testing the hypothesis that immunosuppressive treatment needs to be different depending on these baseline characteristics. While the study can help to identify risk factors for non-response, the underlying mechanisms of neuroinflammation are not yet clarified. Different pathomechanistic steps have been previously reported for neuroinflammation including immune-mediated effects on astrocytes, microglia, oligodendrocytes and neurons e.g. in multiple sclerosis models or graft-versus-host disease affecting the CNS.^9^ The evolution of novel technological advances including genetically modified mouse models in combination with single-cell RNA-sequencing, and high resolution flow-cytometry based analysis of human CNS or PNS tissues, allows an increased depth of immune profiling of neuroinflammation,^10^ which could help to characterize and understand the pathomechanism behind irAEs affecting the PNS and CNS. Immune checkpoint inhibitors used in the clinic are diverse, targeting mainly PD-1, PD-L1, CATLA-4, TIM-3 and LAG-3. The authors found that the type of ICI used was not connected to later responsiveness of the neuroinflammation to immunosuppression.^8^ Conversely, the clinical presentation as myositis and neuromuscular junction disorders were predictive for a better response, suggesting that the biology and pathomechanism may be different in these manifestation compared with other clinical pictures such as limbic encephalitis and sensory neuronopathy, which were connected to low response rates. Currently, the pathomechanism of irAEs affecting the PNS and CNS are not well understood, and future research should analyse in controlled systems how ICI causes inflammation in the PNS and CNS including local immune cell types that may be involved.
